# A strategy to promote the convenient storage and direct use of polyhydroxybutyrate-degrading *Bacillus* sp. JY14 by lyophilization with protective reagents

**DOI:** 10.1186/s12934-023-02173-4

**Published:** 2023-09-15

**Authors:** Su Hyun Kim, Nara Shin, Suk Jin Oh, Jeong Hyeon Hwang, Hyun Jin Kim, Shashi Kant Bhatia, Jeonghee Yun, Jae-Seok Kim, Yung-Hun Yang

**Affiliations:** 1 Department of Biological Engineering, College of Engineering, Konkuk University, Seoul, 05029 Republic of Korea; 2 Institute for Ubiquitous Information Technology and Application, Konkuk University, Seoul, Republic of Korea; 3 Department of Forest Products and Biotechnology, Kookmin University, Seoul, 02707 Republic of Korea; 4 Department of Laboratory Medicine, Kangdong Sacred Heart Hospital, Hallym University College of Medicine, Seoul, Republic of Korea

**Keywords:** Bioplastics, Polyhydroxybutyrate, Biodegradation, Lyophilization, Raffinose

## Abstract

**Background:**

Bioplastics are attracting considerable attention, owing to the increase in non-degradable waste. Using microorganisms to degrade bioplastics is a promising strategy for reducing non-degradable plastic waste. However, maintaining bacterial viability and activity during culture and storage remains challenging. With the use of conventional methods, cell viability and activity was lost; therefore, these conditions need to be optimized for the practical application of microorganisms in bioplastic degradation. Therefore, we aimed to optimize the feasibility of the lyophilization method for convenient storage and direct use. In addition, we incoporated protective reagents to increase the viability and activity of lyophilized microorganisms. By selecting and applying the best protective reagents for the lyophilization process and the effects of additives on the growth and PHB-degrading activity of strains were analyzed after lyophilization. For developing the lyophilization method for protecting degradation activity, it may promote practical applications of bioplastic-degrading bacteria.

**Results:**

In this study, the polyhydroxybutyrate (PHB)-degrading strain, *Bacillus* sp. JY14 was lyophilized with the use of various sugars as protective reagents. Among the carbon sources tested, raffinose was associated with the highest cell survival rate (12.1%). Moreover, 7% of raffionose showed the highest PHB degradation yield (92.1%). Therefore, raffinose was selected as the most effective protective reagent. Also, bacterial activity was successfully maintained, with raffinose, under different storage temperatures and period.

**Conclusions:**

This study highlights lyophilization as an efficient microorganism storage method to enhance the applicability of bioplastic-degrading bacterial strains. The approach developed herein can be further studied and used to promote the application of microorganisms in bioplastic degradation.

**Supplementary Information:**

The online version contains supplementary material available at 10.1186/s12934-023-02173-4.

## Background

Environmental pollution caused by waste discharge is a major global concern [1, 2]. The biggest cause of this problem is the increased use of non-degradable plastics and their accumulated waste [3, 4], resulting in an increased interest in the use of biodegradable bioplastics [5–7]. Bioplastics, produced by microorganisms, sugarcane, and cellulose as replacements for fossil fuels, can be degraded into water and carbon dioxide[8]. Therefore, studies on the microbial production and degradation of bioplastics are being conducted [9, 10]. Among bioplastics, polyhydroxybutyrate (PHB), which can be produced and degraded by microorganisms, has received substantial attention [11–13]. While PHB production is an important research topic, PHB degradation studies are especially crucial. Therefore, increasing number of studies are being conducted on microorganisms capable of degrading PHB [14–16]. Biodegradable bioplastics can be degraded into final products like carbon dioxide and water under mineralization by microorganisms. In this process, the ester bond that makes up the chemical structure of bioplastics can hydrolysis by extracelluar enzymes that are secreted by microorganisms. Their chemica lbond can be broken by esterase family enzymes such as lipase and cutinase. Finally degraded monomers can be utilized as carbon sources by microorganisms [17, 18].

**Fig. 1:**
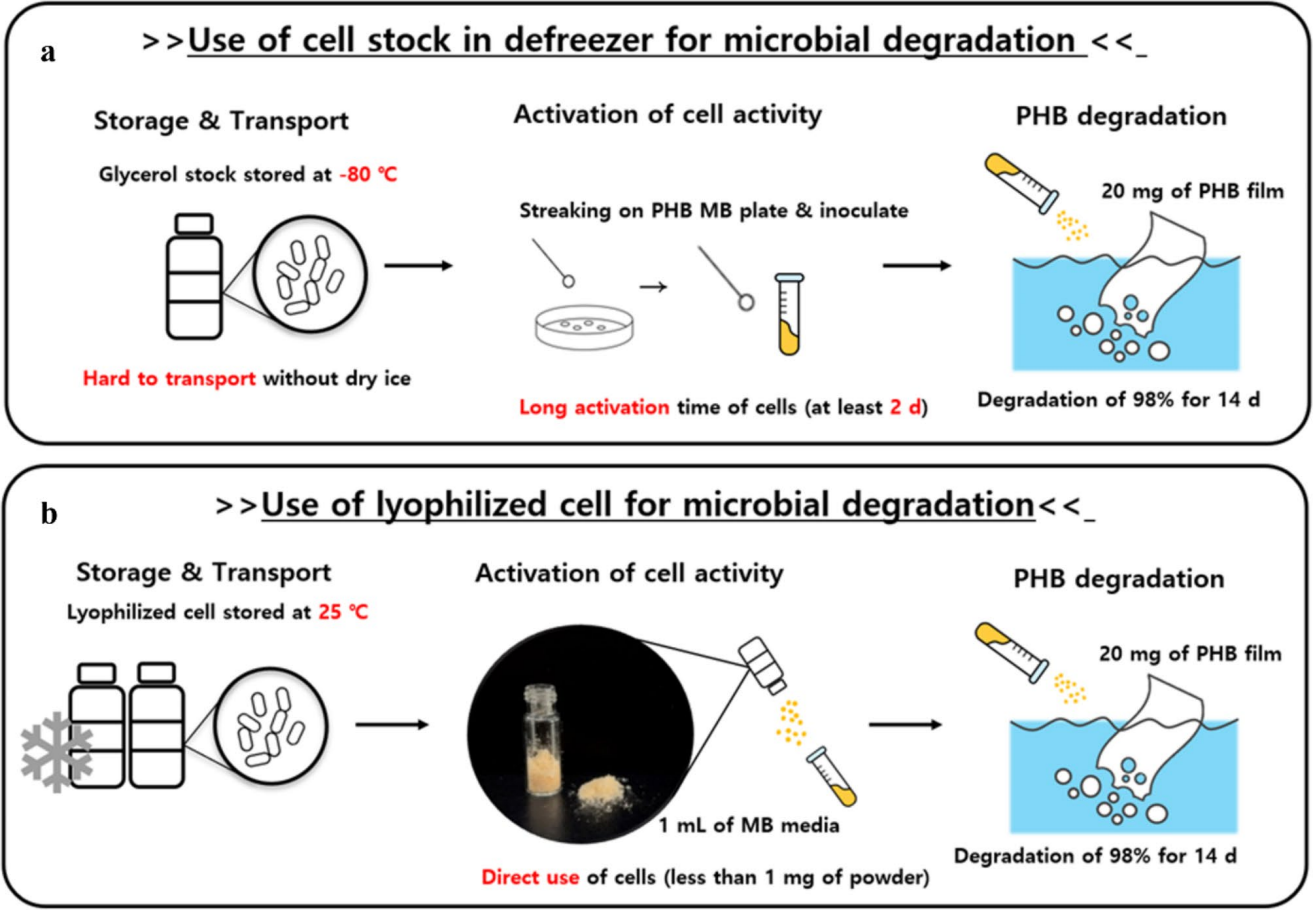
**Comparison of fresh and lyophilized cells.** Compared with fresh cells (**a**), cells lyophilized with 7% raffinose (b) maintained higher levels of cell activity after storage and were more suitable for direct use

To develop PHB-degrading strains for practical applications, several issues need to be addressed. Because microorganisms are highly sensitive to changes in the surrounding environment, employing storage methods that maintain the viability, degradation activity, and stability of the cells is critical [19]. The conventional storage method involves isolating a single colony, adding 20% glycerol to the culture medium, inoculating and culturing the cells for 16–24 h, and storing them at -81 ℃ [20, 21]. However, this method has disadvantages; transport is difficult, and the microbial viability and activity after processing and transport vary with varying bacteria (Fig. 1a). In addition, in order to preserve cellular activity, a laborious process of streaking the colony on solid medium is required, and a long activation time is necessary prior to use.

Lyophilization is a method used to solve these challenges. By storing the microorganism in a powdered form at a low temperature after freeze-drying, the storage period can be extended, and the stability of the strain can be maintained [22–24]. One study reported that freeze-dried microorganisms can be stored for more than 10 years. The target strains were *Brevibacterium* and *Corynebacterium*, and the survival rate for both strains was maintained at approximately 80% after 10 years [25]. In addition, various physiological groups of lyophilized yeast and bacteria were stored at 2–4 ℃ for 50 years, and a high titer of viable cells was maintained, illustrating that lyophilization preserves the viability of bacteria and yeast [26].

However, not all microorganisms can be preserved via lyophilization because the cells experience physical stress during the freeze-drying process [27, 28]. In addition, cells undergo changes in their physical state during lyophilization that result in damage to the membrane lipids and proteins and reduction in bacterial viability. Therefore, various types of protective reagents like sugars have important role in conservation of viability and activity are used during freeze-drying [29]. For example, non-reducing disaccharides, such as trehalose and sucrose, are well-known freeze-drying additives [30] that lower the phase transition temperature of the membrane during the freeze-drying process. In addition, hydrogen bonds are formed between the proteins and phospholipid of the cell membrane and the added sugars during lyophilization, preventing protein denaturation [31]. Also, this process can mitigate mechanical damage due to the ice crystal formation of bacterial cell. Several other types of sugars have also been used as lyophilization reagents [32, 33].

In this study, we aimed to preserve the PHB-degrading activity of *Bacillus* sp. JY14, which was reported to be a superior PHB degrader [34], by selecting and applying the best protective reagents for the lyophilization process (Fig. 1b). The effects of additives on the growth and PHB-degrading activity of *Bacillus* sp. JY14 were studied before and after freeze-drying under different storage conditions. This study is the first to optimize the lyophilization of *Bacillus* sp. JY14. Our findings may promote practical applications of bioplastic-degrading bacteria.

## Results and discussions

### Selection of the incubation period for optimal PHB degradation activity

**Fig. 2:**
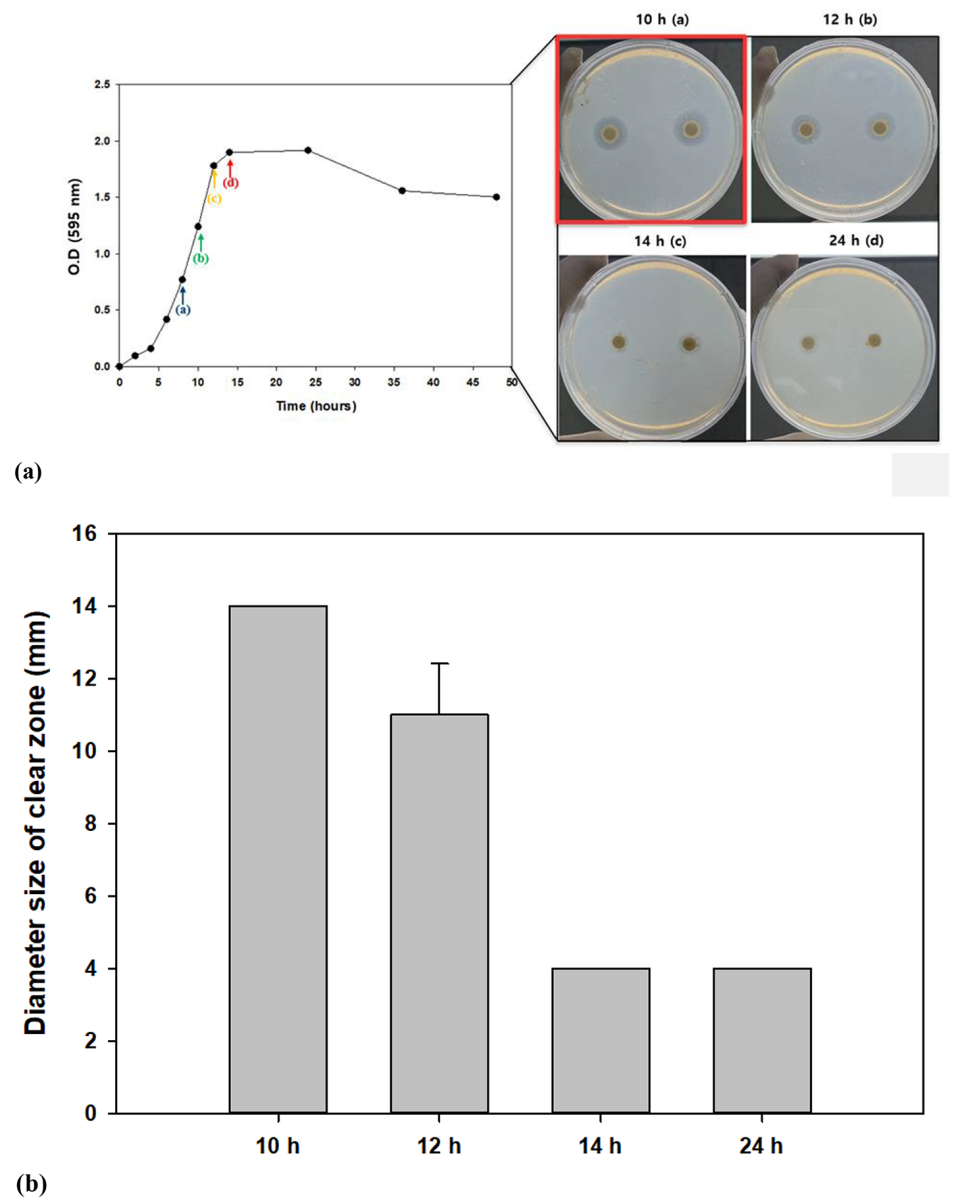
**Confirmation of PHB degradation activity according to growth of*Bacillus*sp. JY14.** (**a**) The largest clear zone appeared after 10 h. (**b**) The size of the clear zone was confirmed by measuring the exact radius of the paper disc. The largest clear zone was observed after 10 h. Data shown are the means and standard deviataions of duplicate experiments

Before lyophilization, the PHB degradation activity of *Bacillus* sp. JY14 was evaluated based on bacterial growth using a clear-zone test to determine the optimal incubation period. Because bacterial activity is not necessarily proportional to the growth curve, we deemed the optimization of incubation period to be essential [35]. Four time points [10 h (a), 12 h (b), 14 h (c), and 24 h (d)] from the start of the log phase to the stationary phase were established, and a clear-zone test was conducted using MB containing a PHB emulsion. The culture medium was collected at each time point. Over 7 d of incubation at 30 ℃, the diameter of the clear zone was measured (Fig. 2a). At all of the time points, a clear zone was observed, but the size differed slightly. Among the four time points, 10 h (a) was associated with the largest and most transparent clear zone (Fig. 2b). However, 14 h (c) and 24 h (d) time points were associated with small clear zones that had poor transparency (Fig. 2a). Therefore, in further experiments, culturing was conducted for only 10 h (a), and cells with the highest level of PHB degradation activity were then harvested.

*Effect of protective reagents on the growth and activity of* Bacillus *sp. JY14 before lyophilization*.

**Fig. 3:**
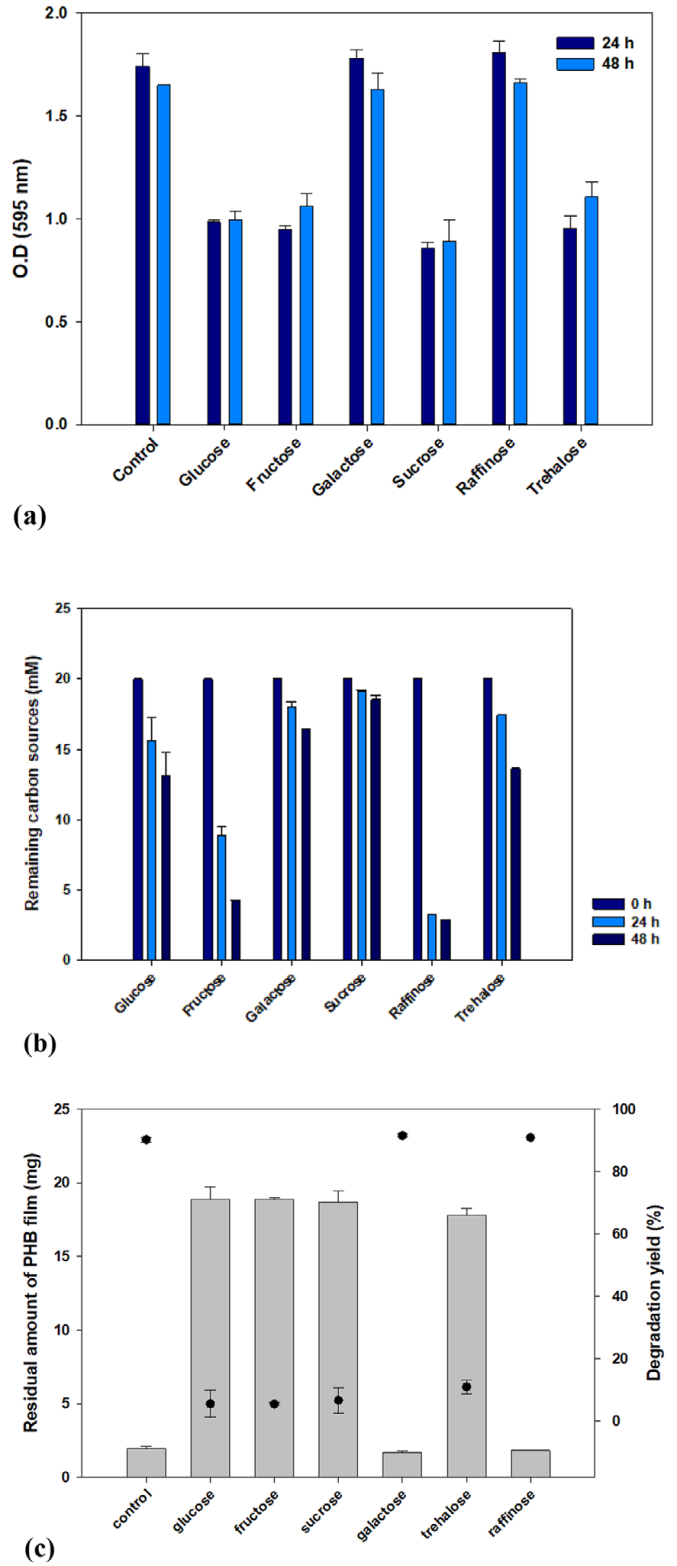
**Effect of carbon sources on*Bacillus*sp. JY14 before lyophilization.** (a) Growth of *Bacillus* sp. JY14, according to carbon sources. (b) Consumption of carbon sources by *Bacillus* sp. JY14 was confirmed. (c) PHB degradation yield was checked according to sugars. Galactose and Raffinose can affect to *Bacillus* sp. JY14 for degrading PHB. Data shown are means and standard deviataions of duplicate experiments

Sugars used as protective reagents can affect the growth and activity of bacterial cells. Therefore, the effect of different protective reagents on *Bacillus* sp. JY14 before lyophilization was evaluated by measuring bacterial growth, sugar consumption, and PHB degradation activity. In liquid culture, 100 mM of each sugar was added to the medium, and the cells were cultured for 48 h at 30 ℃. The cultures incubated with raffinose and galactose exhibited O.D values similar to those exhibited by the control culture to which no carbon sources were added. Conversely, bacterial growth decreased when the other sugars were added to the medium (Fig. 3a). In addition, the consumption of the carbon sources was analyzed using HPLC. *Bacillus* sp. JY14 consumed all of the types of sugars to some extent (Fig. 3b). Fructose and raffinose were effectively consumed but showed no correlations with growth. After 14 d, GC–MS analysis indicated that *Bacillus* sp. JY14 exhibited a good PHB degradation yield (Fig. 3c). *Bacillus* sp. JY14 exhibited a preference for galactose and raffinose consumption; the addition of other sugars did not significantly increase degradation. In particular, raffinose, a trisaccharide composed of galactose, glucose, and fructose, seemed to affect the degradation activity. Consequently, raffinose was deemed the most influential sugar. These results indicate that, when employing protective reagents, their potential effects on bacterial growth and degradation activity should be considered.

### Viability and PHB degradation activity of Bacillus sp. JY14 after lyophilization with protective reagents

After lyophilization, with the addition of sugars as protective reagents, plate-based colony counting methods were used to determine the cell survival rate (Table 1). Cell growth differed depending on the type of sugar added. Raffinose was associated with the highest survival rate (12.1%). Sucrose and trehalose, which are widely known freeze-drying preservatives, were associated with markedly lower survival rates (4.29% and 4.25%, respectively). Fructose was associated with a higher survival rate compared with the control. However, glucose and galactose did not exert a protective effect on cell viability. Therefore, the addition of sugars as protective reagents during lyophilization was confirmed to effectively preserve cell viability. Among the various sugars, raffinose demonstrated the best protective ability.

**Table 1:** Colony-forming units (CFUs) of viable cells and survival rates of *Bacillus* sp. JY14

Protective reagent	Viable cells after lyophilization (${\times 10}^{8}$ CFU/mL)	Survival rate (%)
Control	0.40	0.91%
Glucose	0.80	1.80%
Fructose	1.36	3.09%
Galactose	0.31	0.70%
Sucrose	1.89	4.29%
Raffinose	5.35	12.1%
Trehalose	1.87	4.5%

Next, the PHB degradation activity of the strain was assessed after lyophilization. Degradation activity was evaluated using a clear-zone test (Supplementary Fig. 1). When the cells were lyophilized without a preservative, assessing the size of the clear zone and the growth of the strain around the paper disc was difficult. Larger and more transparent clear zones were observed when sugars were added as lyophilization preservatives. The difference in the size of the clear zones was not substantial, but the increased transparency facilitated evaluations of growth.

**Fig. 4:**
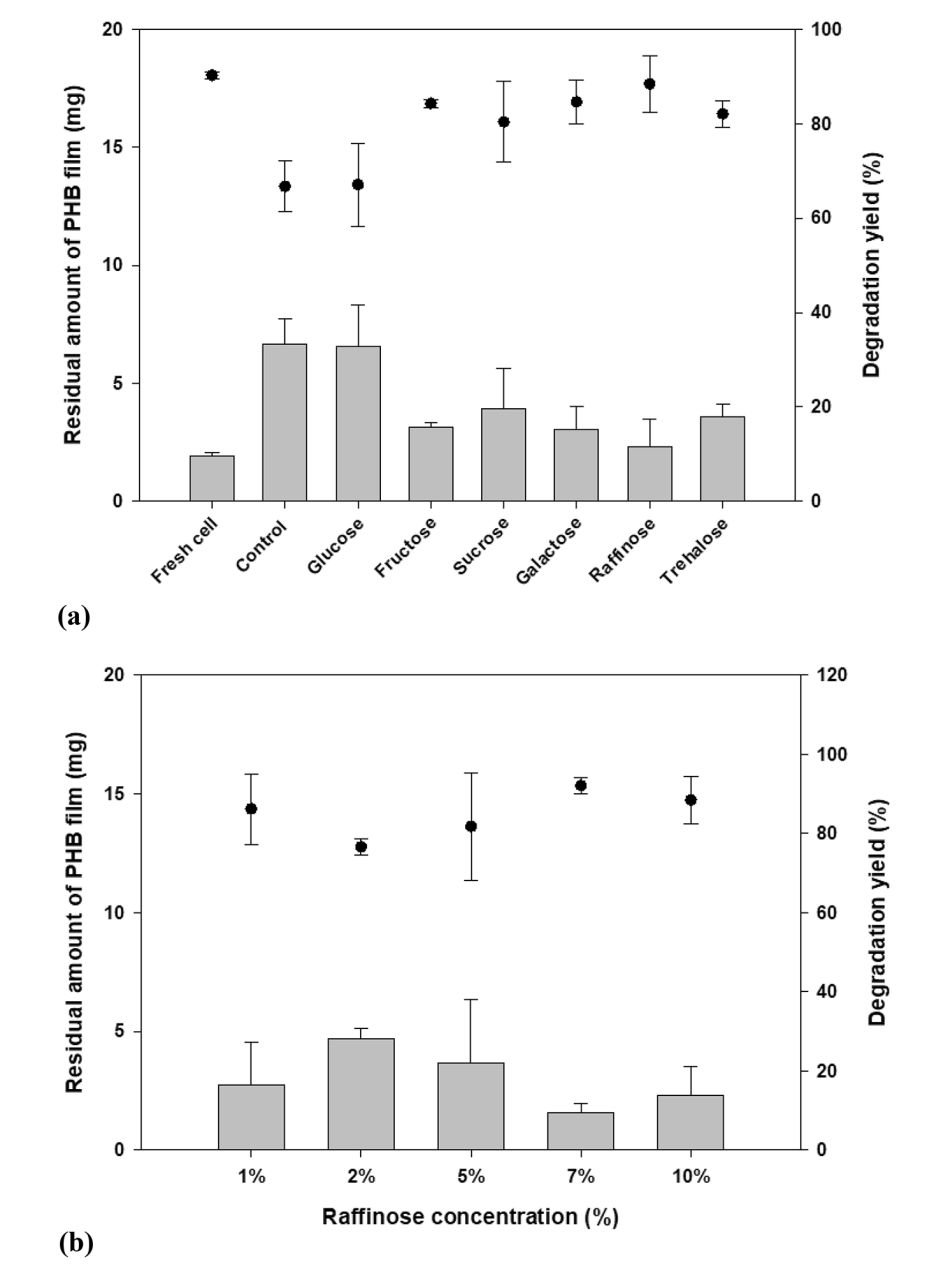
**Biodegradation activity of*Bacillus*sp. JY14 after lyophilization.** (**a**) Polyhydroxybutyrate (PHB) degradation yield was confirmed after lyophilization in the presence of protective reagents. Among the various sugars, raffinose exhibited the highest degradation yield (88.4%). (**b**) A concentration of 7% raffinose produced the highest degradation yield (92.1%); this concentration was selected as the optimal concentration for further experiments. Data shown are the means and standard deviataions of duplicate experiments

In addition, maintenance of PHB degradation activity was tested using liquid culture. Evaluating the degradation of PHB films in a liquid environment is important to determine the extent to which the activity of the strain can be maintained after lyophilization. After lyophilization, the samples were suspended in 3 mL of MB and used as the inoculum for the subsequent liquid culture. The strain was cultured with 20 mg of PHB film at 30 ℃ for 1 wk. After cultivation, the residual films were recovered to analyze the degradation yield, using GC–MS. In the control, which did not contain a preservative, the PHB degradation activity decreased because cell viability and activity were not preserved during lyophilization [36]. Degradation of the PHB film was observed, but the degradation yield was lower than that of the fresh cells. The degradation yield increased when sugars, except glucose, were used as preservatives. Raffinose was associated with the highest degradation yield (88.4%; Fig. 4a).

Based on the above results, raffinose was selected as the preservative for further experiments. The PHB degradation activity changed with the concentration of raffinose (Fig. 4b). A concentration of 7% raffinose was associated with the best PHB degradation activity (92.1%) in liquid culture. Raffinose, a well-known trisaccharide preservative used during lyophilization, was previously confirmed to effectively protect the viability and PHB degradation activity of *Bacillus* sp. JY14 [37, 38]. Hydrogen bonding stabilizes proteins, membranes, and cells during the freeze-drying process. Raffinose is a pentahydrate sugar; therefore, it forms more effective hydrogen bonds with biomolecules than sucrose and trehalose, both of which are well-known protective reagents used in freeze-drying. These properties may facilitate the preservation of *Bacillus* sp. JY14 PHB degradation activity using raffinose [39, 40].

### PHB degradation activity according to storage temperature and period

**Fig. 5:**
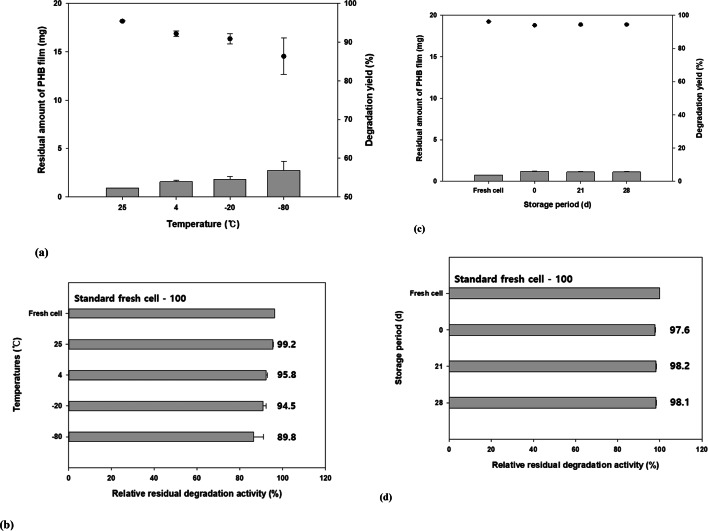
**Effect of storage temperature and period on degradation after lyophilization.** (**a**) Polyhydroxybutyrate (PHB) degradation pattern was assessed at various temperatures. The storage temperature associated with the greatest degradation yield was 25 ℃. (**b**) The preservation of degradation activity was compared at different temperatures; the degradation yield was preserved at over 90% at most temperatures, except for − 80 ℃. (**c**) Polyhydroxybutyrate (PHB) degradation activity was assessed after various storage periods. When samples were stored for 28 d, no major differences were observed compared with control cells. (**d**) Degradation activity was not affected by the storage period. Data shown are the means and standard deviataions of duplicate experiments

The storage environment is critical for preserving the viability and activity of microbes in lyophilized samples. Storage temperature has been reported to affect cell stability and viability. The degree to which cells lose their viability differs with changes in storage temperature [41]. Therefore, we assessed the degradation yield of lyophilized samples maintained in liquid culture at four different temperatures for 7 d and 14 d. Samples stored at 25 ℃ exhibited the highest PHB degradation yield (Fig. 5a). Moreover, the degradation yield decreased with decreasing storage temperature, although the decrease was not dramatic. Lyophilized cells exhibited at least 90% of the degradation activity of fresh cells at all temperatures except − 80 ℃ (Fig. 5b). Therefore, the degradation activity of the lyophilized bacterial samples was not greatly affected by the storage temperature. Although the PHB degradation yield was highest at 25 ℃, -20 ℃ was selected as the storage temperature for further experiments. Storage at 25 ℃ presents issues such as the potential for contamination and the difficulty of maintaining a constant temperature. Samples stored at 4 ℃ are vulnerable to contamination in case of moisture formation, while samples stored 0 ℃ can be affected by fungal contamination.

Many studies have demonstrated the utility of lyophilization as a strategy for maintaining the degradation activity of microorganisms throughout long-term storage. Degradation activity was assessed after 28 d of storage at -20 ℃ (Fig. 5c). Samples stored for 28 d exhibited no changes in the PHB degradation activity; the degradation yield was similar to that of fresh cells. In addition, even as the storage period was increased, the degradation activity of the stored samples was maintained at above 97%, compared to that of fresh cells (Fig. 5d). The degradation rates associated with different storage periods were highly similar (data not shown). Therefore, the lyophilization of this strain with raffinose as a protective agent was highly effective in preserving cell activity for long-term applications.

## Conclusions

Microbial degradation may be a solution to the environmental problems associated with plastics [42]. Therefore, the biodegradation activity of microbial strains found in various environments has been investigated [34, 43, 44]. To broaden the applications of these strains, maintaining their bacterial activity during transport and storage is essential. Lyophilization is one strategy for doing so [25, 45]. In this study, we optimized the preservation and direct use of *Bacillus* sp. JY14 and determined the best protective reagent. Raffinose was identified as the most effective lyophilization preservative. The optimal concentration of raffinose was 7%; this concentration produced the best degradation yield (92.1%), with only small changes observed under different storage conditions. Although raffinose may not be the best protective reagent for every strain, our study demonstrated that protective reagents for preserving biodegrading strains can be identified and successfully applied. From 3 mL of culture medium containing raffinose, we obtained 70 mg of lyophilized sample, and 0.001 mg (70 mg × 0.005/3000) of lyophilized sample was used to degrade 20 mg of PHB film. Therefore, 70 mg of lyophilized sample may be used as an inoculum for degrading 1.4 kg of PHB film. Overall, this study demonstrated the potential of optimized lyophilization in degrading bioplastics. The approach developed herein can be further studied and used to promote the use of microorganisms for practical applications.

### Methods

#### Chemicals used in this study

All chemicals used in this study were of analytical grade. Chloroform was obtained from Fisher Scientific (Seoul, Republic of Korea). Glucose was obtained from Duksan Pure Chemicals (Ansan, Republic of Korea). Fructose, sucrose, glycerol, and dichloromethane (DCM) were obtained from Junsei Chemical Co. (Tokyo, Japan). Galactose was obtained from Daejung Chemicals (Siheung, Republic of Korea). Trehalose and raffinose were obtained from the Tokyo Chemical Industry (Tokyo, Japan). PHB pellets were obtained from Goodfellow Cambridge Ltd. (Huntingdon, UK).

#### Strains for degradation of PHB

This study employed *Bacillus* sp. JY14, which shows the highest degree of similarity (99.02%) with *Bacillus algicola* strain AB423f. This strain, screened from marine soil samples, exhibits a high degree of PHB degradation activity. After 14 d of culture in marine broth (MB), the degradation rate is almost 98% [34].

#### Preparation of Bacillus sp. JY14 and growth curve

In all experiments conducted in this study, *Bacillus* sp. JY14 was inoculated and incubated into MB (Kisanbio, Seoul, Republic of Korea) containing peptone (5.0 g/L), yeast extract (1.0 g/L), ferric citrate (0.1 g/L), sodium chloride (19.45 g/L), magnesium chloride (5.9 g/L), magnesium sulfate (3.24 g/L), calcium chloride (1.8 g/L), potassium chloride (0.55 g/L), sodium bicarbonate (0.16 g/L), potassium bromide (0.08 g/L), strontium chloride (34.0 mg/L), boric acid (22.0 mg/L), sodium silicate (4.0 mg/L), sodium fluoride (2.4 mg/L), ammonium nitrate (1.6 mg/L), and disodium phosphate (8.0 mg/L) at 30 ℃. This strain was pre-cultured in 5 mL of MB for 16 h at 30 ℃.

To determine the growth patterns of *Bacillus* sp. JY14, the pre-cultured strain was inoculated (2%) into MB (50 mL) in a 100-mL flask and incubated for 48 h at 30 ℃. Until growth reached the stationary phase, 200 µL of bacterial culture was used to measure the optical density (OD) at 2-h intervals, using a 96-well microplate reader (TECAN, Switzerland). Aliquots of the cell culture were directly subjected to clear-zone tests at different time points (10, 12, 14, and 24 h) on solid media containing a PHB emulsion. To accurately measure the clear zone, a paper disc (Toyo Roshi Kaisha, Tokyo, Japan) was placed on a plate [46], and 10 µL of pre-cultured cells was inoculated onto the paper disc and cultivated for 7 d at 30 ℃.

To prepare media plates containing PHB, 1 g of a PHB pellet was dissolved in 40 mL DCM in a water bath at 60 ℃. After adding 100 mL of distilled water, 2 mL of 2% Sarkosyl sodium N-lauroyl sarcosinate (NL) solution was added to the boundary between the water and DCM. The mixture was sonicated for 10 min with a 15-s pulse using the Vibra Cell VCX500 (Sonics & Materials, Inc.,Newtown, CT, USA). The amplitude was set to 30% to uniformly mix the contents. After sonication, the solvent was evaporated completely using a stirrer, as per previously described protocols [43, 47]. Then, 1 g/L of the plastic emulsion, uniformly dissolved in the aqueous phase of the solvent, was added to the MB. All mixtures were then autoclaved for 15 min at 121 ℃.

#### Effect of protective reagents on Bacillus sp. JY14

To determine the suitability of various protective reagents, different reagents were compared in terms of their consumption and effects on bacterial growth. Each reagent (2%) was added to 5 mL of MB. *Bacillus* sp. JY14 (2%) was inoculated into the medium and cultured for 48 h on a rotary shaker at 200 rpm. To confirm the effect of the sugars on growth, the OD was measured at 24-h intervals at 595 nm in a 96-well plate. In addition, the consumption of the sugars was analyzed using high-performance liquid chromatography (HPLC). The supernatants were diluted 10-fold with HPLC water and filtered (pore size: 0.2 μm). The HPLC platform (Perkin Elmer, Waltham, MA, USA) was equipped with a refractive index detector and a UV-vis detector. Carbons were separated on an Aminex HPX-87 H column (300 mm × 7.8 mm internal diameter; Bio-Rad, Hercules, CA, USA). The flow rate of the mobile phase was maintained at 0.6 mL/min using 0.004 mol/L sulfuric acid. The oven temperature was set to 60 ℃ [48].

In addition, the effects of the different carbon sources on the PHB-degrading activity of *Bacillus* sp. JY14 were confirmed in liquid culture. The bacterium was cultured for 7 d in a medium containing 20 mg of PHB films. PHB films were prepared using a conventional solvent-cast method [49]. PHB pellets (1 g) were dissolved in 100 mL of chloroform, and the solution was heated in a water bath at 60 °C until the pellets completely dissolved. The solvent containing the dissolved PHB was placed in a fume hood until it completely evaporated and plastic films were formed. These films were cut into 20-mg pieces and sterilized via autoclaving for 15 min at 121 ℃.

#### Preparation of PHB-degrading strains before lyophilization

Obtaining a sufficient amount of *Bacillus* sp. JY14 is necessary, prior to lyophilization The strain (2%) was cultivated in MB (5 mL) for 16 h. To scale up the strain, bacterial cells were inoculated in a 100-mL flask containing 50 mL MB and cultivated in a rotary shaker at 200 rpm. To obtain the most active *Bacillus* sp. JY14 cells, the cells were cultivated for 10 h, based on the findings of this experiment. For lyophilization, cell pellets were obtained by centrifugation at 4 ℃ and 10,000 × *g* for 15 min and washed twice with distilled water. The final O.D values were adjusted to 2.0 to ensure that enough cells were lyophilized for further experiments. Suspended bacterial culture (3 mL) was moved to a 14-mL round tube and centrifuged under the same conditions as mentioned above ; 1 mL of 10% preservation reagent was added to sufficiently suspend the cell pellet. Next, lyophilization was performed at -80 ℃ until the water was completely removed. All preparation processes were conducted in a sterile environment, and all reagents used were sterilized.

#### Viability of Bacillus sp. JY14 after lyophilization

To evaluate the viability of *Bacillus* sp. JY14, the number of cells was measured using the standard pour plate method. After lyophilization, the samples were suspended in 3 mL of MB and diluted serially 10-fold diluted in distilled water. After spreading the samples on MB agar plates, the plates were incubated at 30 ℃ for 24 h. The number of colonies on plates containing 30–300 colony-forming units (CFU)/mL was counted. The survival rate (%) was calculated as follows [50]:

$${\text{Survival rate (\% ) = }}\frac{{{\text{Afterlyophilization}}\,({\text{CFU}}/{\text{mL}})}}{{{\text{Beforelyophilization}}\,({\text{CFU}}/{\text{mL}})}}*100$$

*Confirmation of biodegradation activity of* Bacillus *sp. JY14 after lyophilization*.

After lyophilization, the efficacy of each sugar as a protective agent was assessed in solid and liquid cultures. Lyophilized samples were suspended in 3 mL of MB and immediately used as inocula to evaluate degradation activity in solid and liquid cultures. In solid culture, to determine the size of the clear zone, samples were incubated for 7 d at 30 ℃. Liquid culture, containing 20 mg of PHB film, involved identical conditions at 200 rpm in a rotary shaker. After culturing, the residual films were recovered, washed with distilled water several times and freeze-dried to prepare samples for analysis. The residual PHB films were analyzed using gas chromatography–mass spectrometry (GC–MS), and the degradation yield was calculated [44]. Lyophilized samples were stored at 25, 4, -20, and − 80 ℃ for 7 d to determine the optimal storage temperature. To determine the optimal storage period, samples were stored for 21, and 28 d at -20 ℃.

### GC–MS analysis

The amount of residual PHB and the degradation yield were confirmed using GC–MS. To prepare samples for GC–MS, the culture medium was centrifuged at 10,000 × *g* for 10 min, and residual PBS films were collected and washed several times with distilled water to remove cell debris and residual medium components. The collected samples were lyophilized in Teflon-stoppered glass vials to completely remove water. For methanolysis of PHB films, 1 mL of methanol/sulfuric acid (85:15 v/v) and 1 mL of chloroform were added to the vials, which were then heated for 2 h at 100 ℃. After 2 h, the vials were cooled at 25 ℃ for 10 min, 1 mL of HPLC-grade water was added to the vials, and the samples were vortexed for 1 min. The organic phase was extracted using a pipette and transferred to an e-tube containing anhydrous sodium sulfate to remove water. Samples were filtered (pore size: 0.2 μm), injected into a GC–MS platform (Perkin Elmer, Waltham, MA, USA) equipped with a fused silica capillary column (Elite-5ms; 30 m × 0.25 mm inner diameter × 0.25 μm), and subjected to a linear temperature gradient (50 ℃ for 1 min, increased at 15 ℃/min to 120 ℃ for 2 min, and then increased at 10 ℃/min to 300 ℃ for 10 min). The injector port temperature was 250 ℃. Mass spectra were obtained using electron impact ionization at 70 eV, and scan spectra were obtained within the range of 45–450 m/z. Selected ion monitoring was used for detection and fragmentation analysis of the major products [51]. A calibration curve was obtained to quantify the amount of the residual PHB film.

### Electronic supplementary material

Below is the link to the electronic supplementary material.

Supplementary Material 1

## Data Availability

All data generated and analysed during the current study are available from the corresponding author on reasonable request.
