# Supplementary material for: A strategy to promote the convenient storage and direct use of polyhydroxybutyrate-degrading Bacillus sp. JY14 by lyophilization with protective reagents
Source: Microb Cell Fact. 2023 Sep 15;22:184. doi: 10.1186/s12934-023-02173-4 (PMC10503174; doi:10.1186/s12934-023-02173-4)

**Supplementary Material**

**A strategy to promote the convenient storage and direct use of polyhydroxybutyrate-degrading *Bacillus* sp. JY14 by lyophilization with protective reagents**

Su Hyun Kim^a^, Nara Shin^a^, Suk Jin Oh^a^, Jeong Hyeon Hwang^a^, Hyun Jin Kim^a^,Shashi Kant Bhatia^a,b^, JeongheeYun^c^, Yung-Hun Yang^a,b*^

^a^Department of Biological Engineering, College of Engineering, Konkuk University, Seoul, Republic of Korea

^b^Institute for Ubiquitous Information Technology and Application, Konkuk University, Seoul, Republic of Korea

^c^Department of Forest Products and Biotechnology, Kookmin University, Seoul 02707, Republic of Korea

***Corresponding author**

Prof. Yung-Hun Yang

Department of Biological Engineering, College of Engineering

Konkuk University, Seoul 05029, Republic of Korea.

**E-mail address:**[seokor@konkuk.ac.kr](mailto:seokor@konkuk.ac.kr)

**Phone number:**(+82) 10-9079-9846

**Figure S1**. Confirmation of degradation activity by clear zone test. The PHB degradation activity of the strain was assessed after lyophilization with protective reagents by clear-zone test. Compared with control, larger and more transparent clear zones were observed when sugars were added.


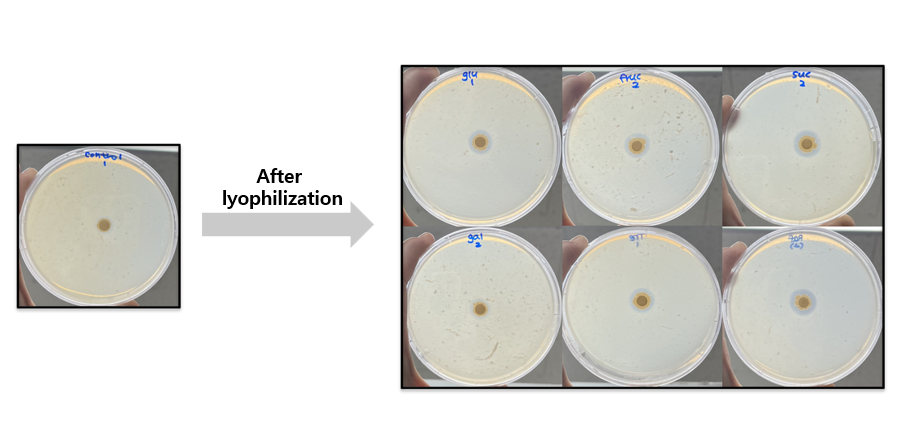


**Figure S2**. A calibration curve obtained from the GC-MS analysis based on the weight of PHB film. This was used to quantify the amount of the residual PHB film.


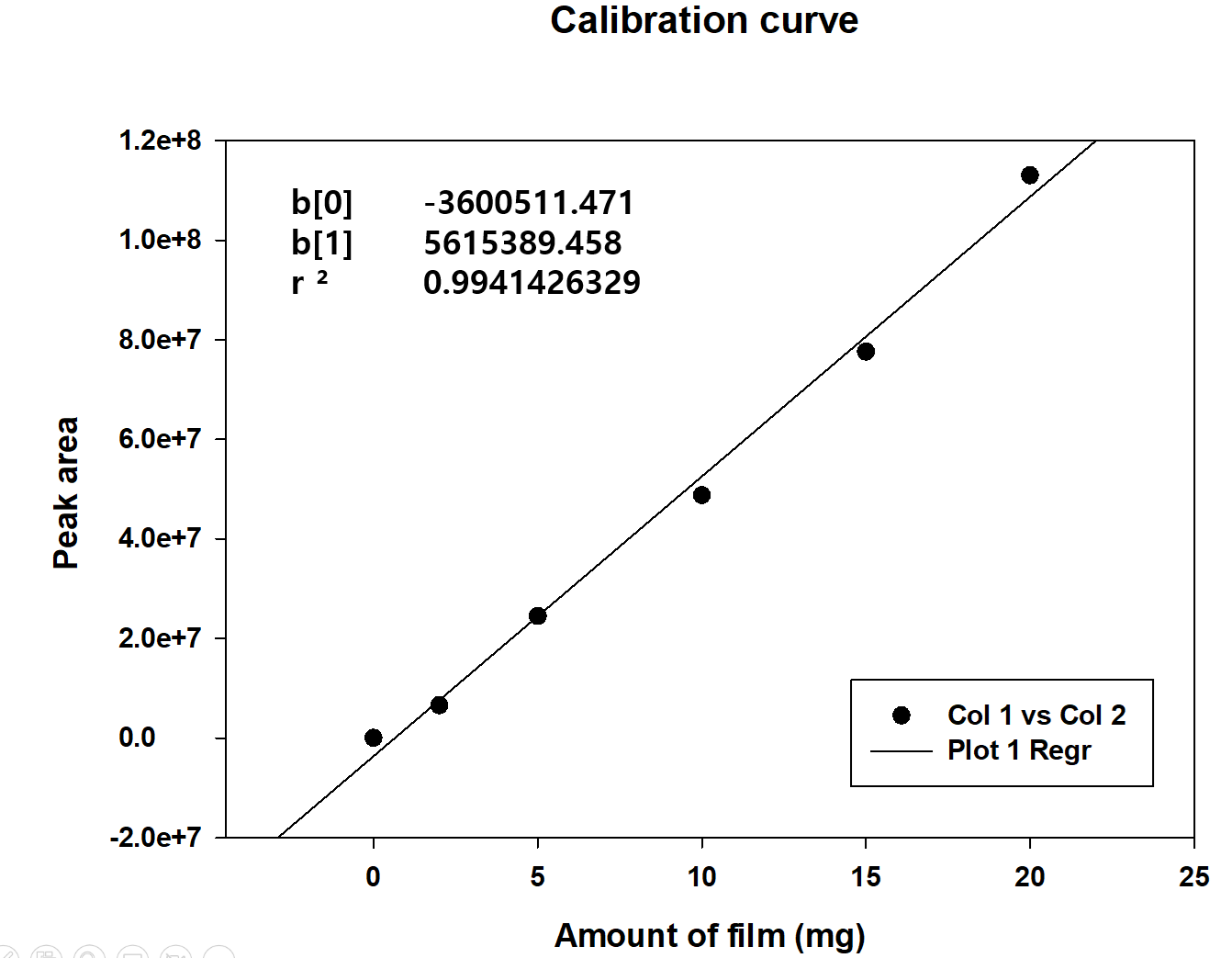

Supplement: Supplementary file 1 — Supplementary Material 1 [file 12934_2023_2173_MOESM1_ESM.docx]
